# Polymer- and peptide-based antiviral surface coatings: progress, mechanistic gaps, and future directions

**DOI:** 10.1128/aem.00638-26

**Published:** 2026-06-17

**Authors:** Umme Laila Urmi, Mark D. P. Willcox

**Affiliations:** 1 School of Optometry and Vision Science, University of New South Wales 7800, Sydney, NSW, Australia; The Pennsylvania State University, University Park, Pennsylvania, USA

**Keywords:** polymer-based coating, peptide-based coatings, surface-mediated virus transmission, antiviral surface coating

## Abstract

Indirect transmission of viruses via contaminated surfaces highlights the need for effective antiviral coatings. Recent advances have led to the development of diverse surface engineering strategies, including organic (polymer- and peptide-based) and inorganic (metal-based) coatings. While polymer and peptide-based systems have been extensively explored in the antibacterial field, their application in antiviral coatings remains underexplored despite their demonstrated ability to reduce viral titers. This minireview provides a mechanistically informed overview of polymer- and peptide-based antiviral surface coatings. We summarize recent studies with a focus on coating materials, methods, physicochemical characterization techniques, target viruses, and antiviral performance. In addition, we critically evaluate key limitations in the field, including the lack of standardized testing protocols, restricted diversity of surfaces and viruses, and insufficient assessment of coating durability and cytotoxicity. Finally, we discuss future directions focused on standardized and rationally designed evaluation frameworks to support the practical translation of antiviral coatings.

## INTRODUCTION

**Fig 1:**
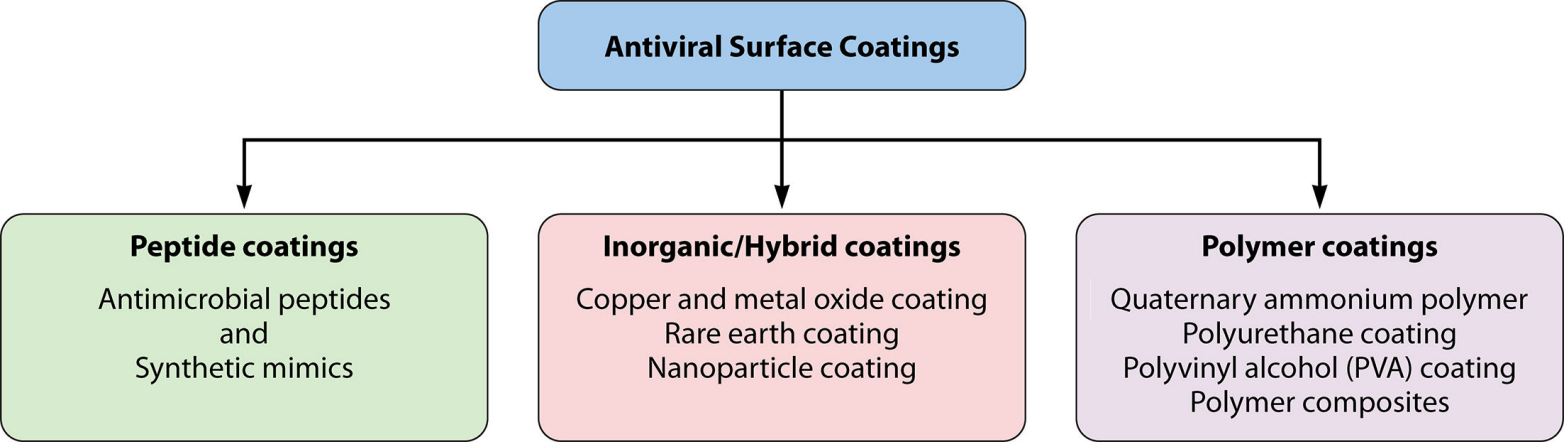
Different types of antiviral coatings.

Virus survival and persistence on contaminated surfaces is a well-recognized route for indirect transmission (1, 2). Viruses can remain infectious on a variety of materials—including plastics, metals, glass, and fabrics—for extended periods depending on environmental conditions and surface properties (3). These contaminated surfaces can therefore act as important reservoirs for virus spread through fomite-mediated transmission. To reduce this risk, surface disinfection is commonly performed using chemical disinfectants. However, this approach relies on continuous and repeated application, which increases operational costs due to labor requirements and may also expose individuals to chemical residues that can cause irritation or allergic reactions (4, 5). These limitations have motivated the development of antiviral surface coatings designed to provide passive and long-lasting protection by continuously reducing viral survival on frequently touched surfaces. Over the past few years, a wide range of antimicrobial surface coatings have been developed using materials such as metals (6), metal oxides (7), polymers (8), antimicrobial peptides (9), and hybrid nanomaterials (10) (Fig. 1).

While many of these coatings were initially designed to target bacterial contamination, the emergence of viral outbreaks has induced growing interest in materials capable of inactivating viruses on surfaces. Despite this interest, antiviral surface coatings remain relatively underexplored compared with antibacterial coatings (11), and a substantial proportion of existing studies focus on metal-based materials.

Interestingly, many compounds reported to possess antiviral activity in solution have not progressed toward therapeutic development due to challenges such as serum binding, cytotoxicity, instability, or poor pharmacokinetic properties. Rather than discarding these compounds, an alternative strategy is to employ them as surface-immobilized antiviral agents, where their activity can be harnessed without the systemic toxicity concerns associated with therapeutic applications.

Among the various material platforms investigated, antimicrobial peptides and polymer-based coatings represent particularly promising approaches for antiviral surface design. Antimicrobial peptides and their synthetic mimics (peptidomimetics) are known for their ability to interact with lipid membranes and disrupt viral envelopes (12, 13), while polymeric materials provide versatile platforms that can be engineered to incorporate antiviral functionality and applied to a wide range of substrates (14). Despite these advantages, relatively few studies have systematically investigated peptide- or polymer-based antiviral surface coatings. Furthermore, the majority of reported antiviral coating studies primarily demonstrate a decrease in viral viability, often measured by plaque assays or 50% tissue culture infectious dose (TCID_50_) assays, without providing detailed insight into the underlying virus inactivation pathway. Key experimental questions frequently remain unaddressed, including the amount of active compound required for complete surface coverage, coating durability under real-world conditions (mechanical abrasion, washing, or disinfection), activity against diverse virus types, and the physicochemical interactions occurring at the virus-surface interface. In addition, comprehensive assessments of cytotoxicity and biocompatibility of coated surfaces are rarely included. Addressing these gaps is essential for the rational development of next-generation antiviral materials.

Despite encouraging antiviral activity, comprehensive mechanistic and comparative evaluations of polymer- and peptide-based coatings remain scarce. This mini review critically examines recent experimental studies focusing specifically on polymer and peptide-based antiviral coatings and highlights key mechanistic and translational gaps.

## POLYMER-BASED ANTIVIRAL SURFACE COATINGS

Polymer-based antiviral surface coatings have been widely explored due to their chemical robustness, versatility, and ease of functional modification. Various chemical strategies have been employed to develop antiviral coatings either incorporating the polymer itself or by incorporating antiviral agents into polymer matrices (Table 1). These coatings had been applied to diverse substrates including glass, plastics, metals, and polymeric materials using methods such as dip-coating, spray-coating, and bulk incorporation during material fabrication.

**TABLE 1:** Polymer-based antiviral surface coatings

Year (reference)	Coating material	Coating method	Surface/substrate	Surface characterization methods^*a*^	Virus tested	Contact time	Durability	Cytotoxicity	Key findings	Mechanism (tested or proposed)
2025 (15)	Polyethylene glycol (PEG) + acrylic resin	Spray-coating	Glass substrate	SEM; EDX; FTIR; TGA	Feline coronavirus	10 min			Maximum 3 log_10_ inhibition (~99.9%) at 20 wt% PEG. Antiviral activity increases with PEG concentration up to optimal level. Higher PEG (25 wt%) reduces performance (poor curing/surface effects). Acrylic alone has no antiviral activity.	
2025 (16)	PVA coating films containing ions from rare earth iodates	Flow-coating	Soda-lime glass	UV-Vis spectroscopy; ATR-FTIR; FE-SEM; SEM-EDX; XRD	Bacteriophage Qβ and U6	2 h; 6 h			Films prepared from Ce (IO3)3 and δ-La (IO3)3 showed strong antiviral activity, whereas Ce(IO3)4 films showed weaker activity	**Proposed:** (i) Rare earth ions neutralizing negative charges of viruses, (ii) oxidizing activity of IO3⁻ ions, and (iii) possible deformation of viral envelope
2024 (17)	LDPE plastic containing rosin	Bulk incorporation	Plastic sheets	SEM; XPS; EDX	African swine fever virus	2 h			Viral titer reduced 1.3 log_10_ after 60 min and ~6 log_10_ suppression after 120 min (no detectable infectious virus); LDPE control reduced <1 log	**Tested:** TEM showed morphological damage of virions after 2 h; suggesting structural disruption
2024 (8)	Copper oxide nanoparticles polymer matrix	Mixed into polymer matrix coating	Aluminum discs	XPS	HCoV OC43	1 h		No significant cytotoxicity reported	Alone weak antiviral effect; strong activity in multi-component coating	**Proposed:** Metal ion interaction with viral proteins and ROS generation
	Cinnamaldehyde polymer matrix	Mixed into polymer matrix coating	Aluminum discs	XPS	HCoV OC43	30 min; 1 h		Negligible cytotoxicity	Strong antiviral activity: synergistic effect when combined with CuO and Poly-ε-lysine	**Proposed:** Interference with viral proteins; docking studies from literature suggest interaction with spike protein
2024 (18)	PHMB	Mixed into polymer matrix then painted and dried	Aluminum discs	XPS	HCoV OC43	30 mins; 1h		Cytotoxic to MRC-5 cells	Strong antiviral effect but cytotoxicity limits application	**Proposed:** Membrane penetration and DNA binding
	Poly(ε-lysine)	Mixed into polymer matrix then painted and dried	Aluminum discs, filter paper, 96-well plates	XPS	HCoV OC43	30 min; 1h		Low cytotoxicity compared with PHMB	Alone modest antiviral effect: strong inhibition when combined with cinnamaldehyde and CuO	**Proposed:** Electrostatic interaction with negatively charged membranes and viral envelope disruption
	LDPE plastic containing rosin	Bulk incorporation	Plastic surfaces	HIM; TEM; cryo-EM; AFM; UV-Vis spectroscopy,	HCoV OC43; SARS-CoV-2	HCoV OC43 (complete decrease in 30 min); SARS-CoV-2 (significant loss in 5 min)	24 h	No cytotoxicity toward MRC-5 cells	Rosin-functionalized plastic rapidly reduced coronavirus infectivity compared with standard LDPE where virus remained infectious up to 24–48 h	**Tested:** Leaching of rosin compounds from the plastic that interact with virus and block infection at the endosomal stage
2023 (19)	Unsaturated polyester of orthophthalic acid with CaCO_3_ filler and MEKP catalyst	Bulk incorporation	Composite tiles/polymer surface	XRF; TGA; SEM-EDS; BET	Human influenza virus A/WKO/46/19; avian coronavirus infectious bronchitis virus (IBV)	Influenza virus: 99.99% inhibition after 60 min contact; IBV: infectivity reduced to 10% after 60 min	2 months		Triclosan-loaded polymer composite showed strong antiviral activity and maintained antimicrobial activity under aging conditions (UV, sunlight, pH).	**Proposed:** Biocidal action of triclosan
2023 (20)	Polyurethane (PU) coating modified with hydantoin precursor 1-(hydroxymethyl)−5,5-dimethylhydantoin (HMD)	Bulk incorporation; immersing partially cured PU films in HMD solution followed by chlorination with sodium hypochlorite	Polypropylene (PP) sheets	ATR-FTIR; TGA, XPS; SEM-EDX; iodometric titration for chlorine content	HCoV-229E; SARS-CoV-2	HCoV-229E: >98% inactivation after 30 min; SARS-CoV-2: complete inactivation after 12 h contact	≥7 days		Strong virucidal activity; coatings remain transparent, stable, rechargeable, and maintain activity over repeated infection cycles	**Proposed:** Oxidative inactivation by N-halamine (N-Cl) groups releasing oxidative chlorine species that oxidize proteins and lipids leading to membrane disruption
2022 (21)	Quaternary ammonium polymers: QA1 (C12-quaternized benzophenone) and QA2 (poly(1-dodecyl-4-vinyl pyridinium bromide))	UV-initiated covalent grafting; immersion in solution	Melt-blown polypropylene (mbPP) and spunbond polypropylene (sbPP) (N95 mask materials)	Zeta potential (surface charge); XPS; SEM	Lentivirus (model), MHV-A59 (murine coronavirus), Suid Herpesvirus (SuHV-1), HCoV 229E	1 h			Up to 4.3 log reduction (MHV-A59) with grafted QA1; ~1.7 log decrease (lentivirus); 3.3–3.7 log attenuation (SuHV-1). Broad activity against enveloped viruses. Covalent grafting more effective than physisorption	**Proposed:** Electrostatic attraction + hydrophobic interaction → membrane disruption of viral envelope
2022 (22)	PDMA with PDA, PEI and in-situ nanosilver	Dip and spray-coating	Polypropylene mask fabric; planar glass substrate	ATR-FTIR; XPS; SEM; ICP-OES (silver ion release); CAM	HCoV-229E	1 log inhibition after 30 min; 4 log reduction after 10 h	4 weeks		Reduces protein adsorption (~90%), prevents microbial adhesion, and rapidly decreases viral titer on coated fabric	**Proposed:** Silver ions (Ag^+^) release from nanoparticles; include Ag interaction with viral surface proteins, binding to viral nucleic acids, or interfering with replication
2021 (23)	Quaternary antimicrobial paint (QAP)	Drop-casting	Polyethyleneimine (PEI)	FESEM; EDX	Influenza virus A/NWS/33 (H1N1)	30 min			Reduced detectable virus plaques (~10,000 viruses killed) compared with uncoated control	**Proposed:** Hydrophobic quaternary polymer interacts with lipid membranes of microbes causing lysis/inactivation
2021 (24)	Quaternary ammonium polymer	Electrostatic spraying	Stainless steel coupons (Type 304)	XRF	HCoV-229E; SARS-CoV-2	2 h		Cytotoxic to Vero E6 cells, but not observed on MRC-5 cells	Reduced virus levels by >99.9% (>3 log) compared with non-coated stainless-steel surfaces	**Proposed:** Disrupt viral envelope lipid bilayers due to cationic functionality
2021 (25)	PHMB	Pad-dry-cure finishing	CVC fabric (50% cotton/50% polyester woven fabric)	Qualitative detection of PHMB using BPB dye complexation	Feline coronavirus	2 h			PHMB-treated fabric achieved 1.22 log drop (~94.01% virus reduction) compared with control fabric	**Proposed**: PHMB may interact with viral capsid leading to virus death
2020 (26)	Cu_2_O microparticles bound with PU	Layer-by-layer coating	Glass slides; stainless steel; doorknobs; credit-card buttons; pens; cart handles	SEM; XPS; EDX; CAM	SARS-CoV-2	1 h	13 days	No toxicity to Vero E6	~99.9% viral titer depletion after 1 h compared with uncoated surfaces	**Proposed**: Cu^+^ ion release → ROS generation damaging viral proteins; direct virus contact with Cu_2_O; inhibition of viral entry
2016 (27)	Sodium pentaborate pentahydrate (SPP) + Triclosan (T)	Immersion of cotton fabric into antimicrobial solution	Cotton textile fabric	ICP-MS; UHPLC/HPLC	HAdV-5; Poliovirus type 1 (PV-1)				Reduced virus titres by 3 log10 (~60% elimination) compared with untreated textiles;	**Proposed**: Inhibit lipid biosynthesis in microbes; Boron compounds: mechanism not fully understood but known antimicrobial activity

^*a*^ AFM, atomic force microscopy; ATR-FTIR, attenuated total reflectance-Fourier transform infrared spectroscopy; BET surface analysis, Brunauer–Emmett–Teller surface area analysis; BPB, bromophenol blue; CAM, contact angle measurement; Cryo-EM, cryogenic electron microscopy; EDX, energy-dispersive X-ray spectroscopy; FE-SEM, field emission scanning electron microscopy; HIM, helium ion microscopy; ICP-MS, inductively coupled plasma-mass spectrometry; ICP-OES, inductively coupled plasma-optical emission spectroscopy; SEM, scanning electron microscopy; SEM-EDS, scanning electron microscopy–energy-dispersive spectroscopy; SEM-EDX, scanning electron microscopy–energy-dispersive X-ray spectroscopy; TEM, transmission electron microscopy; TGA, thermogravimetric analysis; UHPLC/HPLC, ultra-high-performance liquid chromatography/high-performance liquid chromatography; UV-Vis spectroscopy, ultraviolet–visible spectroscopy; XPS, X-ray photoelectron spectroscopy; XRD, X-ray diffraction; XRF, X-ray fluorescence spectroscopy.

For example, polyvinyl alcohol (PVA) coatings incorporating ions derived from rare earth iodates demonstrated antiviral activity against both enveloped and non-enveloped bacteriophages (16), while rosin-functionalized low-density polyethylene (LDPE) plastics showed rapid inactivation of viruses including African swine fever virus and human coronaviruses (HCoV) (17). Several studies had also reported polymer matrices containing antimicrobial additives such as copper oxide nanoparticles (8), cinnamaldehyde (8), poly(hexamethylene biguanide) (PHMB) (18), and poly(ε-lysine) (18), which exhibited antiviral activity against HCoV OC43. In addition, functional polymer coatings such as hydantoin-modified polyurethane (PU) surfaces containing N-halamine groups demonstrated strong virucidal activity against HCoV-229E and SARS-CoV-2 (18).

While some studies focused on coatings containing a single active component, others developed hybrid systems by combining polymers with metal nanoparticles. For instance, poly(N,N-dimethylacrylamide) (PDMA)/ polydopamine (PDA) coatings containing *in situ* generated nano silver were shown to reduce coronavirus titres while also limiting microbial adhesion (22). Earlier studies also reported antiviral activity using quaternary ammonium polymer coatings (24) and PHMB-treated textiles (25), which inactivated influenza virus or feline coronavirus on coated surfaces. Collectively, these reports demonstrated the versatility of polymer-based materials as platforms for antiviral surface coatings.

While these studies demonstrate the potential of polymer-based antiviral coatings, several common trends can be identified (Table 1). In most cases, extensive surface characterization was performed to confirm the successful incorporation of antiviral agents prior to antiviral evaluation (8, 16, 20, 22, 26, 27). However, antiviral efficacy was typically assessed using a limited range of viruses, predominantly enveloped viruses, particularly coronaviruses, with reported loss of virus particles occurring within minutes to hours of contact (8, 15, 18, 20–22, 24–26). Unfortunately, the majority of studies did not include mechanistic investigations to elucidate the underlying basis of antiviral activity. Furthermore, key aspects such as coating durability and cytotoxicity were often not evaluated or reported, highlighting significant gaps that need to be addressed for practical application. These observations highlight the need for deeper mechanistic investigations and more systematic evaluation strategies, which will be discussed in later sections of this review.

## PEPTIDE-BASED ANTIVIRAL SURFACE COATINGS

Despite the well-established antiviral potential of AMPs and their synthetic mimics, relatively few studies had explored their immobilization onto surfaces to develop peptide-based antiviral coatings, and the recently reported examples are summarized in Table 2. Most of the reported systems involve immobilizing the active peptide or peptidomimetic onto glass substrates (9, 28, 29), although other materials had also been investigated. For instance, PDA-mediated immobilization has been used to attach the cationic peptide Mel4 and the peptidomimetic RK758 onto glass surfaces, resulting in reduced infectivity of several viruses including human adenovirus-5 (HAdV-5), murine norovirus-1 (MNV-1), murine hepatitis virus-1 (MHV-1), and influenza A virus H1N1(26). In these systems, minimal compound leaching and high host cell viability were observed, suggesting that immobilized peptide coatings can retain antiviral activity while maintaining good biocompatibility (28).

**TABLE 2:** Peptide-based antiviral surface coatings

Year (reference)	Coating material	Coating method	Surface/substrate	Surface characterization methods^*a*^	Virus	Contact time	Durability	Cytotoxicity	Key findings	Mechanism (tested or proposed)
2025 (28)	Mel4	PDA-mediated immobilization (one-pot coating)	Glass	XPS; ToF-SIMS; CAM	HAdV-5; MNV-1; MHV-1; H1N1	24 h	74 h	≥90% host cell viability (A9, MDCK, Vero, RAW264.7)	Reduced infectivity of non-enveloped viruses: HAdV-5 by 85% and MNV-1 by 90%. No activity against enveloped viruses. Minimal leaching detected.	**Proposed**: Neutralizing capsids of non-enveloped viruses, consistent with activity against these viruses in solution
	RK758	PDA-mediated immobilization (one-pot coating)	Glass	XPS; ToF-SIMS; CAM	HAdV-5; MNV-1; MHV-1; H1N1	24 h	74 h	≥90% host cell viability (A9, MDCK, Vero, RAW264.7)	Reduced infectivity of enveloped viruses, MHV-1 by 68% and H1N1 by 89% on coated surfaces. Minimal compound leaching observed.	**Proposed**: Interaction with viral lipid envelope, consistent with activity against these viruses in solution
2024 (30)	DOPA-Phe(4F)-Phe(4F)-OMe	Self-assembly coating by immersion overnight	PU-based elastomer substrate	SEM; EDS; OM; CAM	T4 bacteriophage	24 h	3 months		Complete viral inactivation (99.9% reduction) of T4 bacteriophage	**Tested:** Antiviral activity attributed to fluorinated phenylalanine residues and peptide self-assembly, while DOPA provides adhesion to the surface
2023 (9)	DOPA-Phe-NH_2_	Drop-casting	Glass	TEM; SEM; AFM; DLS; CD spectroscopy; FT-IR; ATR-FTIR	T4 bacteriophage; Canine coronavirus (CCV)	T4 bacteriophage (24 h); CCV (3 h)		Low cytotoxicity toward HT-29 and A2780 cells	Reduced viral titer by ~4 logs (>99.9% reduction)	**Tested:** TEM analysis showed tail detachment and capsid damage in T4 phage; peptides likely interact with viral structural proteins and disrupt viral structure
	DOPA-Phe(4F)-NH_2_	Drop-casting	Glass	TEM; SEM; AFM; DLS; CD spectroscopy; FT-IR; ATR-FTIR	T4 bacteriophage; CCV	T4 bacteriophage (24 h); CCV (3 h)		Low cytotoxicity toward HT-29 and A2780 cells	Coatings reduced viral titer by ≈4 logs (>99.9% reduction)	**Tested:** TEM analysis showed tail detachment and capsid damage in T4 phage; peptides likely interact with viral structural proteins and disrupt viral structure
2022 (31)	DOPA-Phe(4F)-Phe(4F)-OMe	Compression molding/melt compounding	Polymeric film	HR-SEM; CLSM; FT-IR; XPS; TGA; DSC; CAM	T4 bacteriophage	24 h			Reduced viral activity by 79 ± 9% compared to neat LDPE	**Proposed**: Fluorinated aromatic residues interfere with viral assembly, while catechol groups contribute to antimicrobial activity
2021 (29)	DOPA-Phe(4F)-Phe(4F)-Ome	Drop-casting	Glass	SEM; FT-IR; ATR-FTIR; CAM; XPS	T4 bacteriophage; CCV	T4 bacteriophage (24 h); CCV (3 h)	Immersion in water for 5 min, washing three times with water, and wiping with a wet paper towel at 1 g load. XPS signals did not decrease appreciably. No long-term day-based antiviral durability was reported	CRFK (ATCC CCL-94) cells showed no morphological changes at 10 mg/mL peptide assemblies compared to control; no quantitative cytotoxicity or viability data reported.	Three-layer coating showed the best activity. Against T4, viral titer dropped to 3 ± 6 pfu/mL vs 13,579 ± 2,839 pfu/mL on bare glass; reduction was 3 log vs glass and six log versus stock solution. Against CCV, viral load dropped below detection limit after 3 h. Activity increased with surface coverage.	**Proposed**: Supramolecular/aggregated spherical peptide assemblies rather than the individual components. It also notes fluorinated phenylalanine likely enhances activity and cites phenylalanine aggregates as membrane-disrupting molecules
	DOPA-Phe-Phe-Ome	Drop-casting	Glass	SEM; FT-IR; ATR-FTIR; CAM; XPS	T4 bacteriophage; CCV	T4 bacteriophage (24 h); CCV (3 h)	Immersion in water for 5 min, washing three times with water, and wiping with a wet paper towel at 1 g load. XPS signals did not decrease appreciably. No long-term day-based antiviral durability was reported	CRFK (ATCC CCL-94) cells showed no morphological changes at 10 mg/mL peptide assemblies compared to control; no quantitative cytotoxicity or viability data reported.	Against T4, reduced viral titer to 8029 ± 205 pfu/mL from 13,579 ± 2839 pfu/mL on bare glass, corresponding to about 0.2 log reduction. Against CCV, the three-layer coating reduced viral load below detection limit after 3 h. Overall, it was less active than the fluorinated peptide against T4.	**Proposed**: Supramolecular/aggregated spherical peptide assemblies rather than the individual components. It also notes fluorinated phenylalanine likely enhances activity and cites phenylalanine aggregates as membrane-disrupting molecules
2021 (32)	NH₂-DOPA-(Phe)_2_-(His)_6_-OH	Three-step immersion coating	SiO_2_ surfaces, Ti, Si wafers, mica, and glass substrates	QCM-D; SEM; EDS; AFM; XPS; FT-IR; CAM; UV-Vis spectroscopy	Bacteriophage T4	24 h	Release studies showed Cu(I) and H_2_O_2_ release over 24 h; activity could still occur after months, although reduced		Reduced T4 numbers by >5 orders of magnitude (~100%) compared with uncoated surfaces. The coating is transparent and maintains surface appearance.	**Proposed**: Release of Cu(I) ions and H_2_O_2_, generation of ROS, and membrane damage/genetic material degradation caused by copper species.

^*a*^ AFM, atomic force microscopy; ATR-FTIR, attenuated total reflectance-Fourier transform infrared spectroscopy; CAM, contact angle measurement; CD spectroscopy, circular dichroism spectroscopy; CLSM, confocal laser scanning microscopy; DLS, dynamic light scattering; DSC, differential scanning calorimetry; EDS, energy-dispersive spectroscopy; FT-IR, Fourier transform infrared spectroscopy; HR-SEM, high-resolution scanning electron microscopy; OM, optical microscopy; QCM-D, quartz crystal microbalance with dissipation monitoring; SEM, scanning electron microscopy; SEM-EDX, scanning electron microscopy–energy-dispersive X-ray spectroscopy; TEM, transmission electron microscopy; TGA, thermogravimetric analysis; ToF-SIMS, time-of-flight secondary ion mass spectrometry; UV–Vis spectroscopy, ultraviolet–visible spectroscopy; XPS, X-ray photoelectron spectroscopy.

Several studies have also explored DOPA-containing short peptides that self-assemble on surfaces (9, 29–32), particularly fluorinated phenylalanine derivatives such as DOPA-Phe(4F)-based peptides, which demonstrated strong antiviral activity against T4 bacteriophage and canine coronavirus when deposited on glass or polymeric substrates. Structural analyses in these studies indicated that peptide assemblies could interact with viral structural components, leading to capsid damage or disruption of viral architecture.

In addition, peptide-based coatings have been incorporated into LDPE films or developed through multi-step surface modification strategies that introduce metal-binding motifs capable of generating reactive species. For example, histidine-containing peptide coatings capable of releasing copper ions and reactive oxygen species (ROS) showed substantial reductions in bacteriophage infectivity (32). Together, these findings demonstrated that peptide-based surface coatings can achieve significant antiviral activity through mechanisms including viral capsid disruption (9), interactions with viral envelopes (28), or reactive species generation (29), while maintaining surface stability and low cytotoxicity in few cases (9, 28). However, compared with polymer-based antiviral coatings, the number of experimental studies investigating peptide or peptidomimetic antiviral surface coatings remains inadequate, highlighting an underexplored area in antiviral materials research.

## KEY LIMITATIONS IN CURRENT POLYMER- AND PEPTIDE-BASED ANTIVIRAL SURFACE COATING RESEARCH AND FUTURE DIRECTION

**Fig 2:**
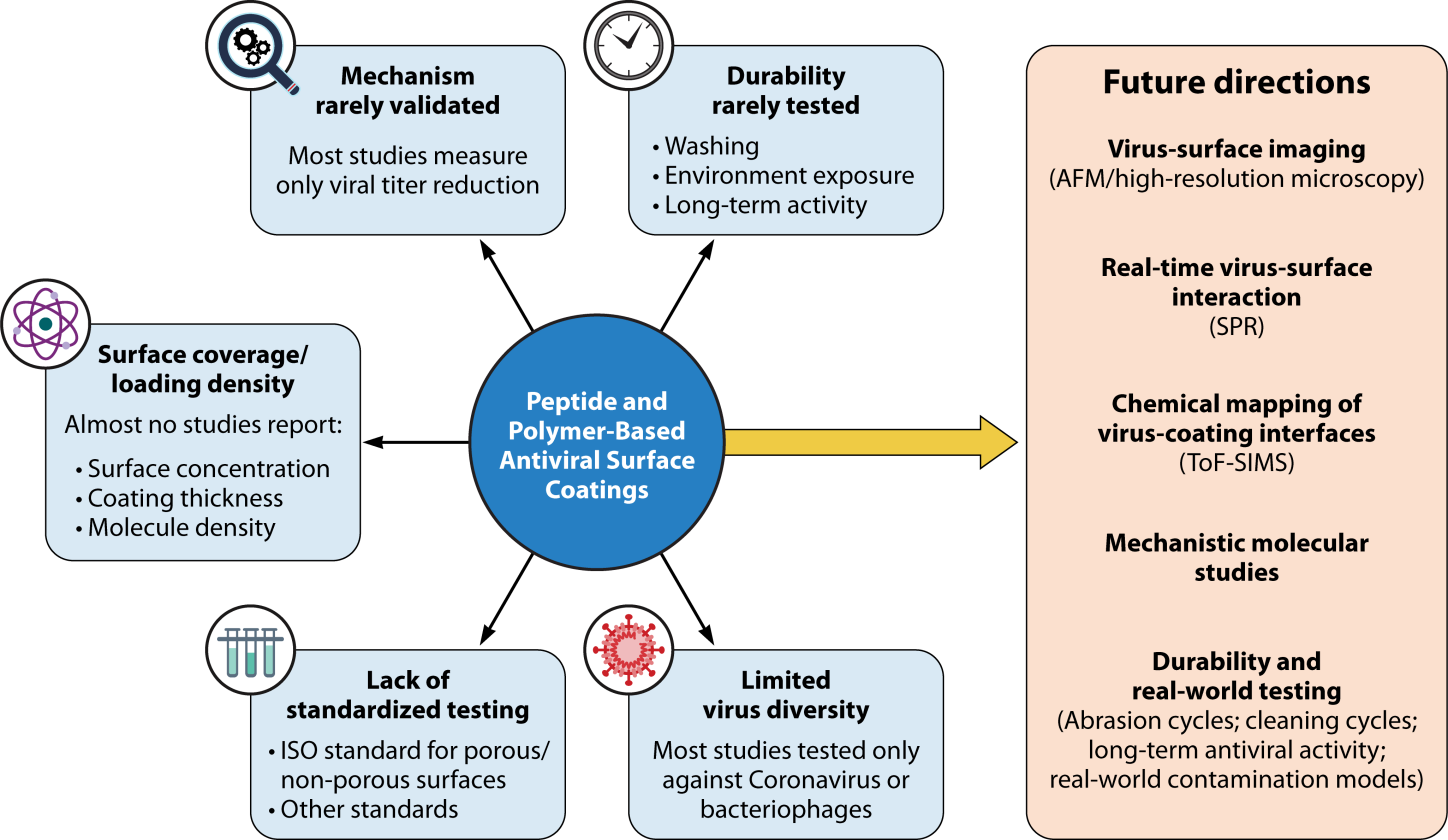
Current gaps and future direction in polymer and peptide antiviral coating research.

In addition to the promising potential of polymer- and peptide-based surfaces to provide protection against indirect viral transmission, the research still faces several critical limitations that stop its meaningful comparison and practical translation (Fig. 2). A critical gap is the inconsistent use of standardized testing methods for antiviral surfaces. Although some studies adopted or followed recognized standards such as ISO 18061, ISO 21702:2019, ISO 22196:2007, or ISO 18184:2019 (8, 16, 18, 19, 25, 28–30, 32), many others (9, 16, 18–20, 30, 32) often use widely different experimental conditions, including viral inoculum levels, contact times, and assay formats. As a result, this research lacks a common benchmark for evaluating efficacy, durability, and comparative studies. This shows a major gap in identifying an ideal coating methods and to moving forward toward real-world applications. In future researchers should prioritize the adoption of standardized testing protocols to evaluate the polymer and peptide based antiviral coatings and use bespoke protocols to highlight other important aspects of their coatings.

Another important limitation is the narrow experimental scope of many studies. Antiviral coatings are often evaluated on a single model substrate (commonly glass or polymer films) and tested against only one or two viruses, typically enveloped viruses such as coronaviruses, influenza viruses, or bacteriophages. Such narrow evaluation makes it difficult to determine whether these coatings would perform similarly on other materials or against structurally diverse viruses, particularly non-enveloped viruses, which are generally more resistant to inactivation. In addition, while most studies perform interfacial analysis to confirm the successful attachment or incorporation of antiviral compounds, quantitative parameters describing the coating itself are rarely reported. For example, information on the amount of active compound required for effective surface coverage, coating thickness, or molecular density on the surface is largely absent. The lack of such quantitative details makes it difficult to establish a clear relationship between coating composition, surface coverage, and antiviral efficacy. To address this limitation, future studies should evaluate coating performance across a broader range of materials, especially those that are believed to contribute to viral spread in places such as homes, hospitals, and industry, and include direct comparative analyses between different coating strategies. In addition, detailed characterization of coating properties such as thickness, surface coverage, and molecular organization is essential, as these parameters critically influence antiviral activity. Importantly, testing should also include a diverse panel of viruses, particularly those with high fomite transmission potential, such as norovirus (33–35).

Similarly, the durability of antiviral coatings under realistic conditions remains poorly investigated. Most reports focus on short-term antiviral activity, while systematic studies evaluating coating stability under repeated contamination cycles, mechanical abrasion, or cleaning procedures are rarely conducted. In future studies, durability tests should be incorporated as a standard requirement, evaluating both long-term antiviral efficacy and potential cytotoxic effects over time.

Cytotoxicity assessments are also minimal, often restricted to a small number of cell lines, providing only partial information regarding coating safety. In parallel, safety evaluation needs to be expanded beyond conventional 2D cell lines to include a wider range of cell types and, where possible, more physiologically relevant systems such as 3D organoids.

**Fig 3:**
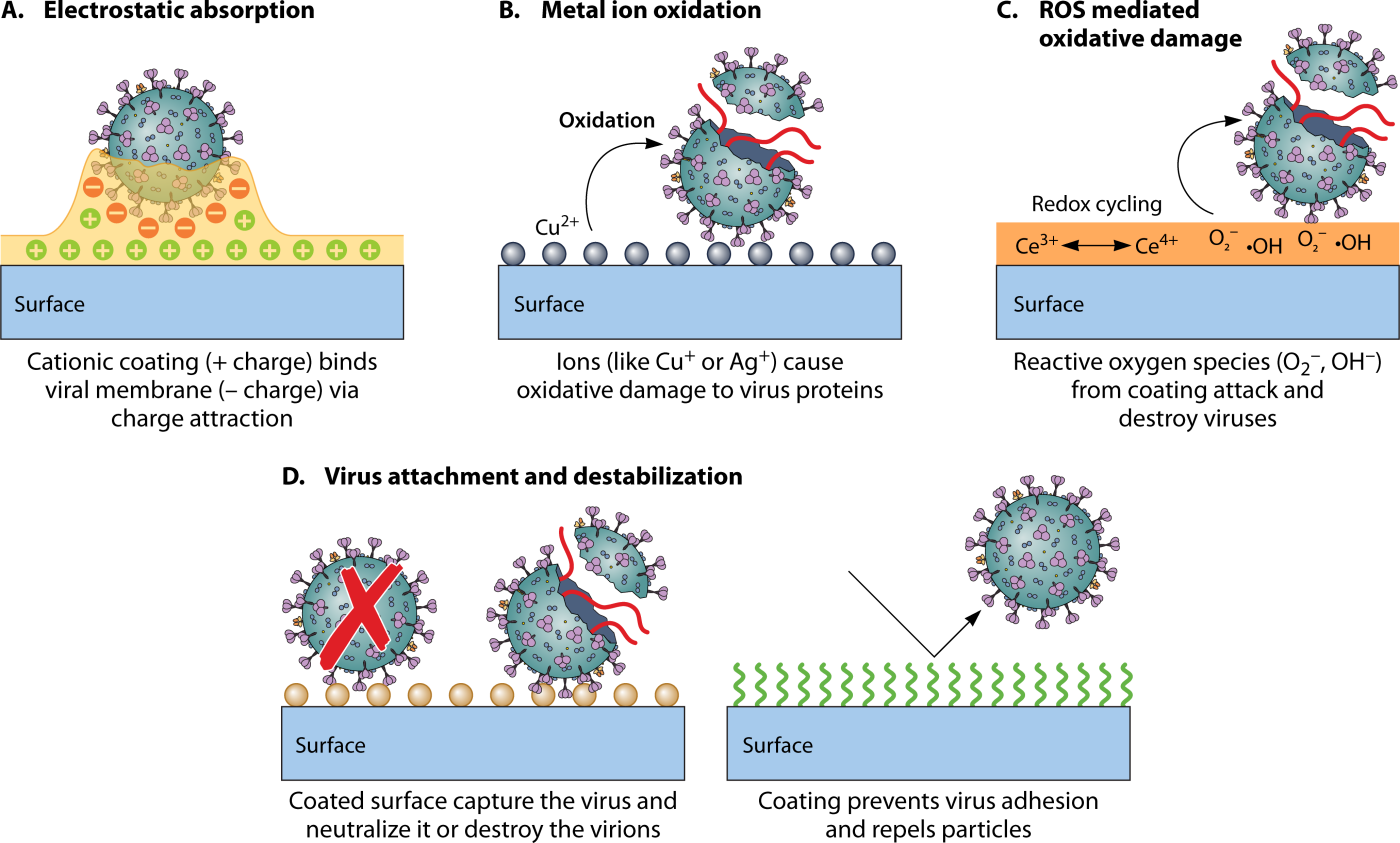
Proposed mode of virus inactivation or inhibition on polymer/peptide-based surface coatings. Virus-surface interactions may involve (**A**) electrostatic adsorption of viral particles to cationic coatings, (**B**) metal-ion-mediated damage to viral proteins or nucleic acids, (**C**) ROS-mediated oxidative damage; (**D**) immobilization and structural disruption of viral envelopes or capsids following surface contact, and antifouling surfaces that prevent viral attachment.

Perhaps the most significant gap is the lack of mechanistic validation of virus-surface interactions. Although many studies demonstrate a decrease in viral infectivity, only a few have directly investigated how viral particles are structurally or chemically altered upon contact with polymer or peptide coated surfaces. However, these have led to the identification of mechanistic pathways to explain virus inactivation on coated surfaces. These include electrostatic interactions between cationic coatings and negatively charged viral membranes (36), metal-ion-mediated protein and genome damage (37), ROS-induced oxidative degradation of viral components (37), and direct capsid destabilization following contact with antiviral surfaces (37) (Fig. 3).

Addressing these limitations will require the integration of advanced analytical approaches capable of probing virus-surface interactions at the molecular and nanoscale levels. Future studies should incorporate techniques such as high-resolution virus-surface imaging (atomic force microscopy, AFM), real-time interaction measurements (SPR), chemical mapping of virus-coating interfaces (ToF-SIMS), and mechanistic molecular analyses (Fig. 3). Such approaches will be essential to fully understand the actual virus-surface interaction pathway of polymer/peptide-based antiviral coating. Overall, these advances will support the practical translation of antiviral surface technologies.

## CONCLUSION

Antiviral surface coatings represent a promising strategy for providing passive protection against viruses on contaminated surfaces. However, this field remains relatively underdeveloped, particularly for polymer- and peptide-based coatings, compared to the more mature antibacterial coating field. These systems offer considerable potential due to their tunable physicochemical properties and ability to provide sustained antiviral activity when immobilized on surfaces. Nevertheless, their translation into practical applications is currently restricted by fragmented study designs and a lack of mechanistic understanding. This minireview highlights key limitations, including the absence of standardized testing protocols, testing on a narrow panel of viruses-primarily coronaviruses and bacteriophages, few mechanistic investigations, inadequate assessment of safety, and insufficient evaluation of long-term efficacy. Moving forward, a standardized and evidence-based approach is essential. This should include the development of unified evaluation frameworks, testing across broader and more representative virus groups, comprehensive coating characterization, and long-term performance assessment. Addressing these challenges will be critical for advancing antiviral coatings from proof-of-concept studies to scalable, real-world infection control solutions.
